# Intracellular ice formation in mouse zygotes and early morulae vs. cooling rate and temperature-experimental vs. theory

**DOI:** 10.1016/j.cryobiol.2016.07.014

**Published:** 2016-10

**Authors:** Bo Jin, Shinsuke Seki, Estefania Paredes, Juan Qiu, Yanbin Shi, Zhenqiang Zhang, Chao Ma, Shuyan Jiang, Jiaqi Li, Feng Yuan, Shu Wang, Xiaoguang Shao, Peter Mazur

**Affiliations:** a Fundamental and Applied Cryobiology Group, Reproductive and Genetic Medicine Center, Dalian Municipal Women and Children's Medical Center, Dalian, Liaoning 116031, China; b Division of Animal Sciences, Bioscience Education and Research Support Center, Akita University, Akita 010-8543, Japan; c Fundamental and Applied Cryobiology Group, Biochemistry, Cellular and Molecular Biology, University of Tennessee, Knoxville, USA

**Keywords:** Intracellular ice formation, Extracellular ice formation, High temperature flasher, Low temperature flasher, Cooling rate

## Abstract

In this study, mature female mice of the ICR strain were induced to superovultate, mated, and collected at either zygote or early morula stages. Embryos suspended in 1 M ethylene glycol in PBS containing 10 mg/L Snomax for 15 min, then transferred in sample holder to Linkam cryostage, cooled to and seeded at 7 °C, and then observed and photographed while being cooled to −70 °C at 0.5–20 °C/min. Intracellular ice formation (IIF) was observed as abrupt ‘‘flashing’’. Two types of flashing or IIF were observed in this study. Extracellular freezing occurred at a mean of −7.7 °C. In morulae, about 25% turned dark within ±1 °C of extracellular ice formation (EIF). These we refer to as “high temperature’’ flashers. In zygotes, there were no high temperature flashers. All the zygotes flashed at temperatures well below the temperature for EIF. Presumably high temperature flashers were a consequence of membrane damage prior to EIF or damage from EIF. We shall not discuss them further. In the majority of cases, IIF occurred well below −7.7 °C; these we call ‘‘low temperature’’ flashers. None flashed with cooling rate (CR) of 0.5 °C/min in either zygotes or morulae. Nearly all flashed with CR of 4 °C/min or higher, but the distribution of temperatures is much broader with morulae than with zygotes. Also, the mean flashing temperature is much higher with morulae (−20.9 °C) than with zygotes (−40.3 °C). We computed the kinetics of water loss with respect to CR and temperature in both mouse zygotes and in morulae based on published estimates of *Lp* and it is *Ea*. The resulting dehydration curves combined with knowledge of the embryo nucleation temperature permits an estimate of the likelihood of IIF as a function of CR and subzero temperature. The agreement between these computed probabilities and the observed values are good.

## 1 Introduction

The major cause of death in cells subjected to freezing is the formation of intracellular ice (IIF). In slow freezing, IIF is avoided by cooling cells sufficiently slowly so that osmotic dehydration results in their water remaining in near chemical potential equilibrium with the outside solution and ice. The faster the cooling, the more the cell water departs from equilibrium, and the more it departs from equilibrium, the more it is supercooled. A supercooled cell will eventually freeze intracellularly at some sub-zero ice nucleation temperature. These procedures can be described quantitatively by four coupled equations which were originally derived by and modified by Mazur [5], [6].

In 1972, Whittingham, Leibo, and Mazur reported the successful cryopreservation of mouse embryos [15]. They found that plots of their survival vs. cooling rate take the form of an inverted U. They hypothesized that the drop in survival above cooling rate of ∼1 °C/min was due to intracellular ice formation (IIF). Subsequently, Leibo et al. [4], made microscope observations on the percentage of mouse oocytes undergoing IIF as a function of cooling rate. The agreement between computed IIF as function of the cooling rates and observed survivals is good for mouse oocytes [4]. As summarized by Mazur [5] such agreement has been found in many other cells [1], [2], [3], [12], [14]. From such agreement has come the important conclusion that IIF is a lethal event and that IIF is responsible for the drop in survival in cells cooled at supraoptimal rates; that is, the right-hand limb of the inverted U. These findings showed that their hypothesis to be correct, but that has never been demonstrated for mouse embryos.

## 2 Material and methods

Our methods have been described in detail in Refs. [9], [11]. Thus, here we give a brief description and details only for those aspects that differed. The procedures for obtaining and manipulating the mouse embryos were carried out under Dalian Medical University and the University of Tennessee Institutional Animal Care and Use Committee protocol 911–0607, approved 2010.

### 2.1 Obtaining mouse zygotes and morulae

Mouse embryos at the one-cell zygote and morula stages were used in the study. Mature female ICR mice were induced to superovulate with intraperitoneal injections of 5 IU of equine chorionic gonadotropin (eCG, Sigma-Aldrich Co. LLC) and 5 IU of human chorionic gonadotropin (hCG; Aldrich Co. LLC) given 48 h later. To obtain the embryos, females were mated with mature males of the same strain immediately after hCG injection. For the collection of one-cell, the oviducts of mated females were flushed with PB1 medium at 24 h after the injection of hCG. For the collection of in vivo developed morulae, the uteri of mated females were flushed with PB1 medium at 76–78 h after the injection of hCG. The embryos were washed and pooled in a culture dish with fresh PB1 medium under paraffin oil until used in the experiments.

### 2.2 Media and linkam sample preparation

For an experiment, embryos were transferred from a PB1 droplet to 1 ml of Dulbecco PBS containing 1.0 M EG and a 0.001% concentration of Snomax (a commercial preparation of freeze-dried *Pseudomanassyringii*, the ice-nucleating bacterium; York Snow Inc., Victor, NY). Snomax is introduced to minimize the supercooling of the suspending medium. Then, 15 min later, a 1.5-μl droplet of this medium was placed in the center of a 50 μm-thick spacer in a Linkam quartz sample cuvette, the embryos were pipetted in a minimum volume to that droplet, and a cover glass applied. The sample cuvette was then inserted in a Linkam BCS 196 cryostage and the freezing-thawing run initiated. The BCS cryostage was attached to a Zeiss bright-field microscope and the zygotes or morulae were observed with an Olympus 20 × long working distance microscope objective.

### 2.3 The linkam cryostage

Using LN_2_ vapor for cooling and electrical resistors for heating, the Linkam cryostage with its associated control hardware and Pax-it software, allows samples to be subjected to sequential ramps in which cooling rate, limiting temperature, holding time, and warming rate can be specified. Our protocol involved five ramps during cooling. The ramps used here are shown in Table 1. The procedure was as follows: the embryos were cooled rapidly to −5.0 °C slowly to −8.0 °C (ramps 1 and 2). External ice formation (EIF) occurred at a mean of −7.7 °C. The sample was then warmed (ramp 3) to −3.2 °C, which is just at the melting point of the medium. At this point, most but not all, of the external ice melted. . The purpose of ramp 3 was to provide time for the external liquid medium, the external ice, and the supercooled water in the cell to come to near equilibrium before recooling began. If ramp 3 was omitted, the observed temperatures of IIF were about 20 °C higher [9]. After 10-sec hold at the end of ramp 3, recooling was initiated in ramp 4. IIF always occurred during ramp 4, manifested by abrupt black flashing of the cell.

## 3 Results

### 3.1 First leg of the triad: modeling and the prediction of IIF in embryos as a function of cooling rate and temperature

The thermodynamic freezing point of most cells (the highest temperature at which ice can co-exist with the protoplasmic solution) is about −0.5 °C. But cells do not freeze even in the presence of external ice unless the temperature falls from 5 to 40 °C below that temperature. By definition, water below its freezing point is supercooled. And supercooled water has a higher vapor pressure, activity, or chemical potential at a given subzero temperature than that of ice or that of water in a solution in equilibrium with ice. The consequence is that as long as the cell contents remain supercooled, the resulting vapor pressure or chemical potential difference will provide a driving force for intracellular water to leave the cell and freeze externally. In other words, the cell will tend to dehydrate during cooling. The rate and extent of that dehydration depends primarily on two variables. One is the inherent permeability of the cell to water; i.e., the hydraulic conductivity, *L*_p_. The other is the cooling rate. For a cell of given *L*_p_, the slower it is cooled, the more it is able to lose sufficient water to remain in near chemical potential equilibrium with external ice and solution, and conversely, the faster it is cooled, the less it is able to dehydrate and the more its water will become supercooled as the temperature falls.

This qualitative description can be described quantitatively by four coupled equations [6], [9]. The first equation relates the rate of loss of cytoplasmic water to the difference in chemical potentials of intracellular and extracellular water expressed as a vapor pressure ratio; i.e.

(1) $$dV/dt=\left(L_{\text{p}}ART\;\ln\;P_{\text{e}}/P_{\text{i}}\right)/v_{\text{W}}$$

where *V* is the volume of cell water, *t* is time, *L*_p_ is the permeability coefficient for water (hydraulic conductivity), *A* is the cell surface area, *R* the gas constant (μm^3^ atm/deg mole), and *v*_w_ the molar volume of water. The ratio *p*_e_/*p*_i_ is the external and internal vapor pressures of water, this ratio is less than 1 because the intracellular water is supercooled and the vapor pressure of supercooled water is greater than that of ice or of water in a solution in equilibrium with ice. The change in this vapor pressure ratio with temperature can be calculated from a second differential equation derived from the Clausius-Clapeyron relation and Raoult's law:

(2) $$d\;\ln\left(p_{e}/p_{i}\right)dT=L_{f}/RT^{2}-\left[n_{2}v_{w}/\left(n_{2}v_{w}\right)V\right]dV/dT$$

Here, *n*_2_ is osmoles of solute in the cell and *L*_f_ is the molar latent heat of fusion of ice. Time and temperature are related by the cooling rate, which, if linear, is given by:

(3) dT/dt=B

Finally, the hydraulic conductivity, *L*_*p*_, decreases with falling temperature. If it is assumed tofollow an Arrhenius relation, its value at a given absolute temperature, *T*, is given by:

(4) $$Lp=L_{pg}\;\exp\{-E_{a}/R^{\prime }\left[\left(1/T\right)-\left(1/T_{g}\right)\right]\}$$

where the subscript g refers to the value at a given reference temperature (usually 20 °C or 0 °C), and *R*′ is the gas constant, here expressed in the units cal/deg mol. *E*_*a*_ is the activation energy of *L*_p_ in cal/mol. *R*, *R*′, *L*_f_, and *v*_w_ are constants, the values of which are given in Table 2. The values of *A*, *n*_2_, *L*_pg_, and *E*_*a*_ are constant for a given cell but differ in different cells. *E*_a_ may also be a different value below 0 °C than above 0 °C. The values in Table 2 are for mouse zygotes and morulae. Knowledge of *L*_pg_, *Ea*, *n*_2_, and *A/V* (the surface to volume ratio of the isotonic cell) permit one the compute the volume of cell water (and the extent of supercooling) versus subzero temperature and cooling rate. Eqs. (1), (2), (3), (4) are solved by the Runge–Kuttamethod [5], assuming *L*_f_ to be constant. Actually, as discussed in the appendix in Refs. [6], it decreases with falling temperature, but the effect on the kinetic curves is small.

**Fig. 1:**
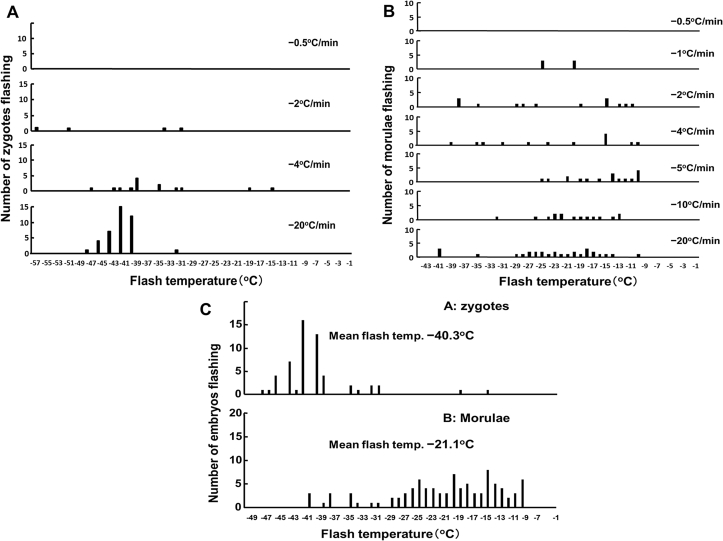
Frequency distribution of the flash or IIF temperature or zygotes (A, C data from Table 3) and morulae (B, C data from Table 4) suspended in 1 M ethylene glycol/PBS at various cooling rates.

The results of such computations are shown in Fig. 1A for mouse zygotes and Fig. 1B for morulae. Both plot the volume of cell water during cooling as a fraction of the volume of water in the unfrozen cell for a range of cooling rates. Both depict an equilibrium curve (*E*_*q*_). It is the volume of water that a cell has to possess to remain in chemical potential equilibrium with external ice; that is, the volume of water in a cell that is cooled infinitesimally slowly. The curve is generated by the equation below, where V′ is the fractional water volume, Vi is the initial water volume, and Mi is the initial osmolality [5].

$$V^{\prime }=V/V_{i}=v_{w}M_{i}\times 10^{-15}/\exp\left[L_{\text{f}}/R\left(1/T-1/273\right)\right]-1$$

### 3.2 Calculated kinetics of water loss with respect to cooling rate and temperature

**Fig. 2:**
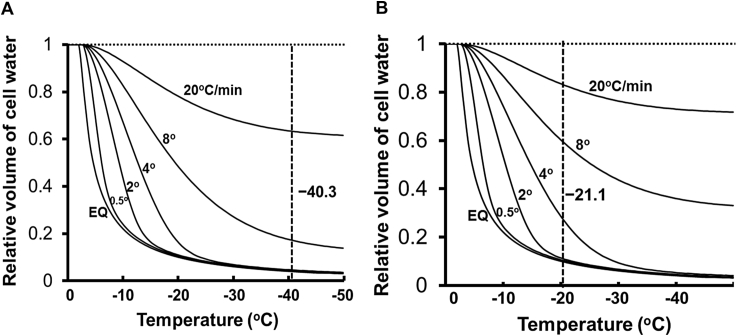
Kinetics of water loss from mouse zygotes (A) and morulae (B) during freezing in 1 M ethylene glycol/PBS. The curve labeled EQ is the volume of cell water required to keep it in chemical potential equilibrium with the external ice and water. This is equivalent to the volumes of water in cells cooled infinitely slowly. The curves were computed from the equations given in the text. Values of the several constants and adjustable parameters are given in Table 2.

In Fig. 2, the higher the cooling rate, the more the curves shift to the right of the equilibrium curve. The number of degrees the curve is shifted is the number of degrees the cell water is supercooled at given temperatures. The vertical lines at −40.3 °C and −21.1 °C [Fig. 1, Fig. 2] represent the temperature at which a supercooled embryo becomes capable of being nucleated.

Note that the water volumes of zygotes merge with the EQ curve well above −40.3 °C when they are cooled at 0.5–4 °C/min (Fig. 2A), but remain above the equilibrium volume when they were cooled at 8 or 20 °C/min. Our prediction would that such zygotes would be undergo IIF when cooled at ≥8 °C/min, but would not undergo IIF when cooled at 4°C/minor slower. In the case of the morulae (Fig. 2B), the diving line would be between 2 °C/min and 4 °C/min those cooled≥4 °C/min would undergo IIF below −21.1 °C; those cooled at≤2 °C/min would not undergo IIF.

One can also express these results in terms of the calculated cell water contents at the cell nucleation for cells cooled at various rates. For zygotes cooled at 8 and 20 °C/min, their fractional water contents at −40.3 °C are17% and 63%, respectively, while the equilibrium water content is 4% (Fig. 2A). For morulae cooled at 4 and 8 °C/min, their factional water contents at −20.9 °C are 29% and 59%, respectively (Fig. 2B), which also are far above the equilibrium value of 10% at that temperature (Fig. 2B).

### 3.3 Second leg of the triad: the observed temperature if IIF as a function of cooling rate and temperature

As stated in Methods, our criterion of IIF was dark flashing. With morulae, we observed two types of flashing; namely, high temperature and low temperature. Extracellular freezing (EIF) occurred at a mean of −7.7 °C. In morulae, about 33% (54/164) turned dark within 1 °C of the EIF temperature (Table 4, column 4). We define these as “high temperature” flashers. The other 67% underwent IIF more than 1 °C below the EIF temperature (column 6), and we refer to those as “low temperature” flashers.

In case of embryos, low-temperature flashers were the only ones observed in embryos undergoing IIF. Fig. 1 shows the frequency distribution of the number if zygotes undergoing low-temperature as a function of cooling rate and temperature. Several conclusions are evident. First, no zygotes underwent IIF when cooled at 0.5 °C/min and only 24% when cooled at 2 °C/min (Table 3). In contrast, 88% and 100% underwent IIF when cooled at 4 °C/minor 20 °C/min. Second, the distribution of the temperatures at which low temperature flashing occurred in individual zygotes was much narrower in those cooled at 20 °C/min than in those cooled at 4 °C/min. Third, the mean temperatures for IIF were −43.3, -34.8, and −42.9 °C for zygotes cooled at 2, 4, and 20 °C/min, respectively, or an overall mean of −40.3 °C (Fig. 2A).

Morulae behaved differently. As shown in column 4 of Table 4, 27.8% of them underwent high temperature flashing (i.e. flashing within 1 °C of EIF). The temperature distribution of the 72.2% that flashed at low temperature is shown in Fig. 1B. The main similarity between their response and those of the zygotes (Fig. 1A) is that no flashing occurred in samples cooled at 0.5 °C/min. but in the morulae cooled at higher rates, the responses differed in an important aspect from those of zygotes. The mean flash temperature of morulae −21.0 °C, (Column 7 of Table 4) that was twenty degrees higher than that of the zygotes (Column 5 of Table 3). Another difference is that, the temperature distribution of flashing with a cooling rate of 20 °C/min was much broader with morulae than with zygotes.

### 3.4 Comparison between cryomicroscope observations and computations on the percentage of mouse embryos undergoing IIF as a function of cooling rate and temperature

**Fig. 3:**
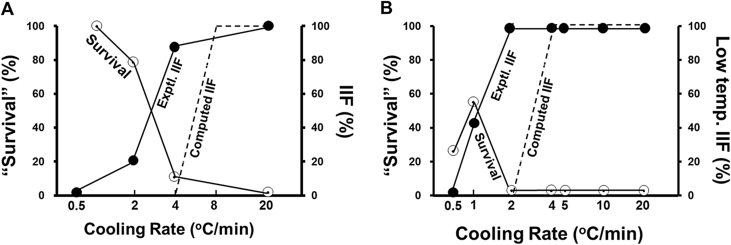
Comparison between the percentages of mouse zygotes (A) and morulae (B) that underwent intracellular freezing and the percentages that computed as a function of cooling rate to −70 °C.

Our criterion of IIF is the “flashing” of a zygote or morula during subzero cooling. In zygotes, the percentages of exhibiting IIF increased from 0% to 24%–88% and to 100% as the cooling rate was increased from 0.5 °C/min to 2, 4, and 20 °C/min (Table 3). These results are shown by the closed symbols and solid line in Fig. 3A. The analogous data for percent flashing in morulae comes from column 6 in Table 3. The normalized percentages of low-temperature IIF as a function of cooling rate are plotted in Fig. 3B by closed circles. The normalized values are obtained by subtracting out those that underwent high-temperature flashing; i.e., the normalized % low temperature flashing = # low flashing morulae/(Total # morulae - #high temperature flashing morulae) x 100.

The main difference between the results Fig. 3 is that maximum IIF in the zygotes is attained when they are cooled at 4 °C/min whereas in morulae, it occurs at 2 °C/min.

The dashed line in Fig. 3 shows the computed probabilities of IIF as a function of cooling rate. They were determined from the kinetic shrinkage cruves in Fig. 2.

If for a given cooling rate, the calculated water volume of the zygote or morula has returned to the equilibrium value before the temperature has dropped below the observed ice nucleation temperature, we assume that IIF can not occur with further cooling. And assign a probability of 0% to IIF. On the other hand, if the calculated cell water volume exceeds the equilibrium value at the cell ice nucleation temperature, we assign a probability of 1 to IIF. The differences in the cooling rate calculated to produce a given % IIF is about 1.5–2 times that of the experimentally observed cooling rate. That is, in the zygotes, 50% IIF occurs at about 1.5 °C and 2.5 °C/min, respectively.

### 3.5 Statistics

Error figures in tables and error bars in graphs are standard errors (standard deviations of the mean). Tests of significance were carried out by one-way ANOVA using Graphpad Software's Instat, V. 3.02 followed by the Tukey–Kramer Multiple Comparison Test.

## 4 Discussion

Since Whittingham, Leibo, and Mazur hypothesized that the drop in survival above cooling rate of ∼1 °C/min was due to intracellular ice formation (IIF) [15]. And subsequently Leibo et al., found microscope observations on the percentage of mouse oocytes undergoing IIF as a function of cooling rate [4]. The purpose of this study was to investigate IIF is responsible for the drop in survival in other developmental stages of mouse embryos by experimental observations and theoretical analysis.

### 4.1 Effect of cooling rate on IIF and IIF temperature

In the present study, two types of IIF were observed. Presumably high temperature flashers were a consequence of membrane damage prior to EIF or damage from EIF. Our aim is to focus on the low temperature flashers.

A major factor determining whether or not cells survive freezing to low subzero temperatures is the rate at which they are cooled. Commonly, plots of their survival vs. cooling rate take the form of an “inverted U” [8]. It was reported that survival of cells was as function of cooling rate in mouse marrow stem cells [3], yeast [2], mouse sperm [1], and human red cells [12], [14]. Since IIF is a lethal factor during cooling. In present study, whether zygotes or morulae, the percentages of IIF rise sharply as the cooling rate increases [Table 3, Table 4; Fig 3]. That is, the likelihood of IIF was a function of cooling rate not only in mouse oocytes [4] but also in mouse embryos.

Several investigators have reported that the IIF temperatures of mammalian oocytes and embryos rise with increasing cooling rate [4], [7], [10], [13]. However, Mazur et al. [9], find that not to be the case in mouse oocytes frozen in 1 M EG/PBS over a 10-fold range of cooling rates (5–50 °C/min). But the IIF temperatures are different between the zygotes (−40.3 °C) and morulae (−20.9 °C). The results are in agreement with those previously reported at the cooling rate of −20 °C/min [11].

### 4.2 Computed likelihood of IIF from predictions modeling, and the occurrence of IIF observed by microscope

We computed the kinetics of water loss with respect to cooling rate and temperature in both mouse zygotes [Fig. 2A] and in morulae [Fig. 2B] based on published estimates of the Lp and it's Ea [6]. The predictions from the modeled curves fall into two groups, depending on cooling rate. The first prediction is that zygotes cooled at 0.5, 2, and 4 °C/min [Fig. 2A] should not undergo IIF and morulae cooled at 0.5, and 2 °C/min [Fig. 2B]. The reason is that they have shrunken to equilibrium. They will by definition not be supercooled and, therefore, will not freeze internally. This prediction agrees with microscope observations.

The second group of predictions is that cells cooled at 8 or 20 °C/min in zygotes [Fig. 2A] and 4, 8, or 20 °C/min in morulae [Fig. 2B] should undergo IIF with near certainty. Because they cross the nucleation temperature of −40.9 or −20.9 °C containing far more cell water than is the case at equilibrium. This prediction also agrees with microscope observations.

From the results of the present study, it is clearly that IIF is a functional of cooling rate in mouse embryos based on not only prediction but also experimental observations.

## Conflict of interest

None.

## Statement of funding

This work was supported by NIH grant R01-OD011201 and National Natural Science Foundation of China (NSFC) 81571498.

## Figures and Tables

**Table 1:** Linkam cryostage cooling and warming ramps for zygotes and morulae frozen in 1 M ethylene glycol/PBS.

Ramp no.	Rate (°C/min)^a^	Limit (°C)	Hold (sec)	Capture intervals	Comments
1	−20	−5	0	30	Cooling
2	−2	−8.0	0	10	Cooling; EIF^b^
3	+2	−3.2	10	10	Warming; partial thawing
4	−0.5 to −20	−70	0	10	Cooling; IIF^c^
5	+20	+20	60	30	Warming and thawing

a Cooling is indicated by negative rates; warming by positive rates.

b EIF refers to extracellular ice formation.

c IIF refers to intracellular ice formation.

**Table 2:** Parameters and constants for calculating water loss of mouse embryos during freezing at various rates.

Item	Symbol	Value	Units
Osmolality of cytoplasm^a^	*M*_*i*_	1.34	Osmolal
Freezing point of cytoplasm^a^	*T*_f_	270.6	K
Initial vol. of cell water at *T* = *T*_f_	*V*_i_	1.88 × 10^5^	μm^3^
Osmoles of solute in cell	*N*_2_	2.52 × 10^−10^	Osmoles
Hydraulic conductivity	*L*_p_	0.43@20°C^b^	μm/min/atm
		0.42@22°C^c^	
Activation energy of *L*_p_ (*E*_a_)	*E*_*a*_	13^b^, 13.5^c^	kcal/mol
Area of cell protoplast	*A*	1.84 × 10^4^	μm^2^
Gas constant	*R*	82.057 × 10^12^	μm^3^ atm/(mol deg) cal/mol deg
	*R*′	1.987	
Molar volume of water	*V*_w_	18 × 10^12^	μm^3^/mol
Molar heat of fusion	L_f_	5.95 × 10^16^	μm^3^ atm/mol
Cooling rates	*B*	0.5–20	°C/min
Temperatures	*T*	293–223	K

a After equilibrium with 1 M EG.

b The Mean *L*_p_ and *E*_a_ of mouse zygotes reported by Leibo (1980).

c The Mean *L*_p_ and *E*_a_ of mouse morulae computed from Edashige et al. (2006).

**Table 3:** Percentage of zygotes flashing as a function of cooling rate and flash temperature.

Cooling rate (°C/min)	n	No flash % (n)	Low flash % (n)	Low flash temperature (°C)
0.5	18	100 (18)	–	–
2	20	80 (16)	20 (4)	−43.3 ± 6.4^a^
4	17	11.8 (2)	88.2 (15)	−34.8 ± 2.5^a^
20*	40	0	100 (40)	−42.9 ± 0.4^a^

*Data from Seki and Mazur, Biology of Reproduction 82, 2010.

a Values with different superscripts within the same column are significantly different (one-way ANOVA, P < 0.05).

**Table 4:** High temperature flashers vs. low temperature flashers for mouse morulae at various cooling rates.

Cooling rate (°C/min)	n	No flash % (n)	High flash % (n)	High flash temperature (°C)	Low flash % (n)	Low flash temperature (°C)
0.5	15	26.7 (4)	73.3 (11)	−7.9 ± 0.12	0 (0)	–
1	18	55.6 (10)	11.1 (2)	−7.9 ± 0.10	33.3 (6)	−22.5 ± 1.12
2	23	0	39.1 (9)	−8.0 ± 0.15	60.9 (14)	−23.8 ± 2.84^a^
4	23	0	39.1 (9)	−8.2 ± 0.12^a^	60.9 (14)	−21.5 ± 2.69
5	23	0	21.7 (5)	−7.9 ± 0.04	78.3 (18)	−15.1 ± 1.21^b^
10	20	0	25.0 (5)	−7.8 ± 0.14	75.0 (15)	−20.1 ± 1.35
20*	42	0	31.0 (13)	−7.7 ± 0.08^b^	69.0 (29)	−23.6 ± 1.48^ac^
Mean			34.3 27.8 (1–20 °C/min)	−7.9	68.8 (2−20 °C/min)	−21.1

*Data partly from Seki and Mazur, Biology of Reproduction 82, 2010.

a–c Values with different superscripts within the same column are significantly different (one-way ANOVA, P < 0.05).
